# Relationship between coronary artery disease with dyslipidaemia and trace mineral intake: a cross-sectional analysis of the Shika study

**DOI:** 10.1017/jns.2024.26

**Published:** 2024-09-23

**Authors:** Kei Kimura, Fumihiko Suzuki, Hiromasa Tsujiguchi, Akinori Hara, Sakae Miyagi, Takayuki Kannon, Keita Suzuki, Yukari Shimizu, Thao Thi Thu Nguyen, Koji Katano, Atsushi Asai, Tomoko Kasahara, Masaharu Nakamura, Chie Takazawa, Koichiro Hayashi, Toshio Hamagishi, Aki Shibata, Takehiro Sato, Akihiro Nomura, Tadashi Konoshita, Yasuhiro Kambayashi, Hirohito Tsuboi, Atsushi Tajima, Takayuki Kobayashi, Hiroyuki Nakamura

**Affiliations:** 1 Department of Public Health, Graduate School of Advanced Preventive Medical Sciences, Kanazawa University, Kanazawa, Japan; 2 Department of Cardiovascular Medicine, Shizuoka Medical Center, Shimizu-cho, Japan; 3 Department of Hygiene and Public Health, Faculty of Medicine, Institute of Medical, Pharmaceutical and Health Sciences, Kanazawa University, Kanazawa, Japan; 4 Community Medicine Support Dentistry, Ohu University Hospital, Koriyama, Japan; 5 Advanced Preventive Medical Sciences Research Center, Kanazawa University, Kanazawa, Japan; 6 Innovative Clinical Research Center, Kanazawa University, Kanazawa, Japan; 7 Department of Biomedical Data Science, School of Medicine, Fujita Health University, Toyoake, Japan; 8 Faculty of Health Sciences, Department of Nursing, Komatsu University, Komatsu, Japan; 9 Faculty of Public Health, Haiphong University of Medicine and Pharmacy, Hai Phong, Vietnam; 10 Department of Bioinformatics and Genomics, Graduate School of Advanced Preventive Medical Sciences, Kanazawa University, Kanazawa, Japan; 11 Department of Cardiovascular Medicine, Kanazawa University Graduate School of Medical Sciences, Kanazawa, Japan; 12 Third Department of Internal Medicine, University of Fukui Faculty of Medical Sciences, Fukui, Japan; 13 Department of Public Health, Faculty of Veterinary Medicine, Okayama University of Science, Imabari, Japan; 14 Graduate School of Human Nursing, The University of Shiga Prefecture, Hikone, Japan

**Keywords:** Angina pectoris, Lipoproteins, Logistic models, Myocardial infarction, Triglycerides, Zinc

## Abstract

Although the relationship between dyslipidaemia (DL) and coronary artery disease (CAD) or between trace minerals intake and CAD is well known separately, the exact nature of this relationship remains unknown. We hypothesize that the relationship between trace mineral intake and CAD may differ depending on whether or not the individual has DL. The present study analysed the relationships among trace mineral intake, DL, and CAD in middle-aged and older adults living in Shika town, Ishikawa prefecture, Japan. This study included 895 residents following the exclusion of those with genetic risk carriers for familial hypercholesterolemia. Trace mineral intake was evaluated using the brief-type self-administered diet history questionnaire. Interactions were observed between DL and CAD with zinc (*p* = 0.004), copper (*p* = 0.010), and manganese intake (*p* < 0.001) in a two-way analysis of covariance adjusted for covariates such as sex, age, body mass index, and current smokers and drinkers. Multiple logistic regression analysis showed that zinc (odds ratio (OR): 0.752; 95% confidence interval (CI): 0.606, 0.934; *p* = 0.010), copper (OR: 0.175; 95% CI: 0.042, 0.726; *p* = 0.016), and manganese (OR: 0.494; 95% CI: 0.291, 0.839; *p* = 0.009) were significant independent variables for CAD in the dyslipidaemic group. The present results suggest that DL with a low trace mineral intake is associated with CAD. Further longitudinal studies are required to confirm this relationship.

## Graphical abstract

**Figure:**
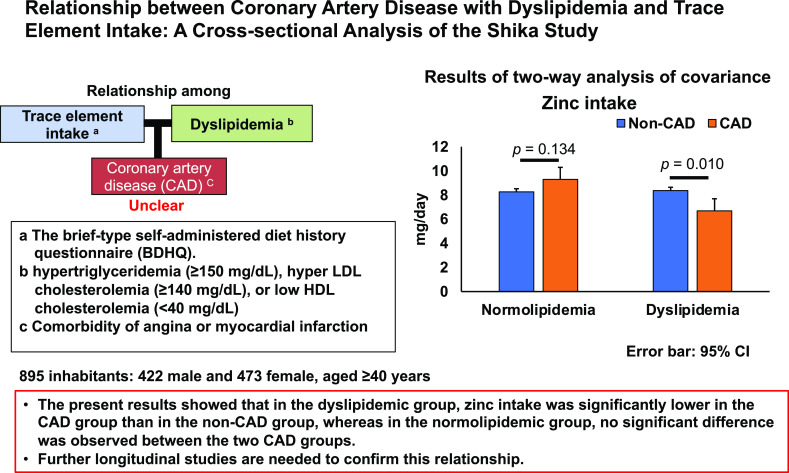

## Introduction

Coronary artery disease (CAD) is estimated to affect 1655 per 100000 individuals worldwide (126 million people) and accounts for 9 million deaths annually. It is regarded as one of the leading causes of death and disability globally.^(1)^ Risk factors for CAD include diabetes, hypertension, smoking, dyslipidaemia (DL), obesity, and social stress.^(2)^ DL is a well-established risk factor for CAD and is estimated to account for more than 50% of CAD cases worldwide.^(3)^ The diagnosis of DL is based on high-density lipoprotein cholesterol (HDLC), low-density lipoprotein cholesterol (LDLC), and triglycerides (TG).^(4)^

One of the nutrients related to serum lipids is trace minerals, such as zinc,^(5,6)^ copper,^(7,8)^ and manganese.^(9,10)^ Zinc plays an essential role in many biochemical pathways as a cofactor for more than 300 enzymes, such as the expression of several genes, and in immune function regulation in many types of cells.^(11,12)^ A cohort study by Chen *et al.* ^(13)^ showed that an adequate nutritional zinc intake was associated with lower CAD mortality. Moreover, a systematic review by Ranasinghe *et al.* ^(6)^ revealed that zinc supplementation significantly reduced total cholesterol, LDLC, and triglycerides. Regarding the relationship between copper and lipids, a cross-sectional study by Song *et al.* ^(14)^ demonstrated that high serum copper levels were associated with elevated serum concentrations of total and HDLC. Additionally, a review by Blades *et al.* ^(15)^ found that an increased copper intake ameliorated the detrimental effects of a high-fat diet. Regarding manganese, a cohort study by Meishuo *et al.* ^(9)^ showed a relationship between its high intake and a lower risk of CAD. Even though the relationship between these trace minerals and serum lipids or CAD has been examined separately, the exact nature of this relationship remains unknown.

Although the relationship between DL and CAD has been extensively examined, the effects of trace mineral intake on this relationship remain unclear. We hypothesize that the relationship between trace mineral intake and CAD may differ depending on whether or not the individual has DL. Therefore, the present study analysed the relationships among trace mineral intake, DL, and CAD in middle-aged and older adults living in Shika town, Ishikawa prefecture, Japan.

## Materials and methods

### Study population

**Fig. 1.**
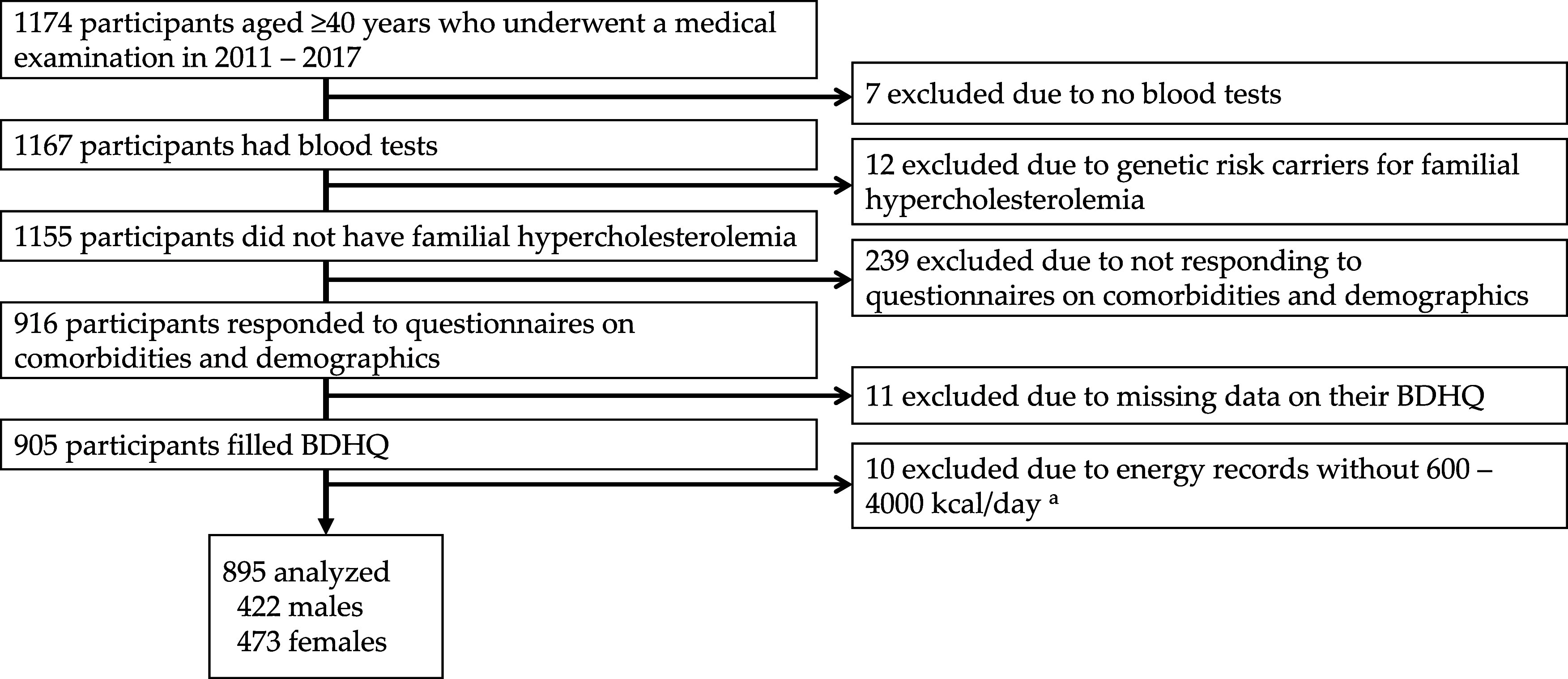
Participant recruitment chart. ^a^ This range is due to the following reasons: less than 600 kcal/day is equal to half the energy intake demanded by the lowest physical activities; more than 4000 kcal/day is equivalent to 1.5-fold the energy intake needed for the medium physical activities. Abbreviations: BDHQ, Brief-type Self-Administered Diet History Questionnaire.

We used data from the Shika study.^(16–18)^ Participants were recruited between October 2011 and January 2017. The target inhabitants were residents living in 4 model districts (Tsuchida, Higashimasuho, Togi, and Horimatsu districts) in Shika town, Ishikawa prefecture, Japan. The population of Shika town was 18786 as of November 2022, with an average age of 55.8 years.^(19)^ The main industries in Shika town are manufacturing and services, with an ageing population of 39.9% at the time of the survey, making it a typical Japanese community in a superaged society. Written informed consent was obtained from all 4546 participants. One thousand and one hundred and seventy-four people who underwent a medical examination agreed to participate in this study. Of these, 7 had no blood tests, 12 had at least one of risk alleles for two single nucleotide polymorphisms (SNPs) which have been frequently observed among Japanese familial hypercholesterolemia (FH) patients as described below, 239 had not responded to the comorbidity and demographics questionnaire, 11 had not responded to the brief-type self-administered diet history questionnaire (BDHQ),^(20,21)^ and 10 had a daily intake outside the range of 600–4000 kcal on the BDHQ and, thus, were excluded. Figure 1 shows inclusion criteria. In total, 895 participants (422 men and 473 women with mean (standard deviation, SD) ages of 62.32 (11.19) and 62.91 (11.41) years, respectively) were included in the analysis. The present study was conducted following the Declaration of Helsinki, and the protocol was approved by the Ethics Committee of Kanazawa University (No. 1491; Dec. 28, 2021). Informed consent was obtained from all participants involved in the study.

### Evaluation of trace mineral intake

Zinc, copper, and manganese intakes were investigated using the BDHQ, which uses an analytical algorithm to estimate the intake of adult Japanese nutrients from the daily food consumed habitually, excluding supplements. The BDHQ collects data on the previous month and seasonal food intake from a structured four-page dietary intake questionnaire. The filled-out questionnaire is sent to the DHQ Support Center (Tokyo, Japan), where a computer system algorithm estimates the food consumed by the participant as the intake of each nutrient. The validity of the BDHQ has been demonstrated in previous studies.^(22,23)^

### Evaluation of DL

Blood samples were collected during annual medical check-ups in the Shika study, mostly between December and January. DL was defined as hypertriglyceridaemia (≥150 mg/dL), hyper LDLC (≥140 mg/dL), or low HDLC (<40 mg/dL),^(4)^ and/or receiving DL medication (*n* = 123). Genetic data, which were obtained in the previous study,^(24)^ were utilized to identify the risk carriers for familial hypercholesterolemia (FH). Briefly, genomic DNA was isolated from peripheral blood samples using QIAamp DNA Blood Maxi Kit (Qiagen, Hilden, Germany) according to manufacturer’s instructions or consigning the company specialized in clinical laboratory testing (SRL, Inc., Tokyo, Japan). Genome-wide SNP typing was performed using the Japonica Array v2^(25)^ (TOSHIBA Co., Ltd. Tokyo, Japan). We defined individuals carrying at least one of the risk alleles for rs564427867 or rs879255211, which have been frequently observed among Japanese FH patients,^(26)^ as genetic risk carriers and excluded them from the statistical analysis. For these two SNPs, deviations from the Hardy–Weinberg equilibrium were not observed (*p* = 1.00).

### Evaluation of CAD and demographic data

A self-administered questionnaire was used to obtain data on comorbidities and lifestyle behaviors. CAD was defined as having a comorbidity of angina or myocardial infarction. Hypertension and diabetes questionnaire items were used as comorbidities associated with DL. Demographic data included age, sex (1: man, 0: woman), body mass index (BMI), smoking status (1: current smoker, 0: non- or ex-smoker), and drinking status (1: current drinker, 0: non-drinker or occasional drinker).

### Statistical analysis

Participants were classified into the normolipidaemic and dyslipidaemic groups or the non-CAD and CAD groups. For statistical analyses, IBM SPSS Statistics version 25 for Windows (IBM, Armonk, NY, USA) was utilized. The Student’s *t*-test was applied to examine the relationships between continuous variables, while the chi-squared test was adopted to investigate those between categorical variables. A two-way analysis of covariance (ANCOVA) was performed to analyse the main effects and interactions between the two lipidaemic groups and two CAD groups with trace mineral intake. Adjustments were performed for the following confounding factors: age, sex, BMI, current drinkers, and current smokers. To confirm the trace minerals that showed an interaction between the two lipidaemic groups and two CAD groups, a multiple logistic regression analysis stratified by the lipidaemic status with CAD as the dependent variable was performed. Variables were chosen using the forced entry method. The significance level was set to 5%.

### Sample size

We used the free software, G-power, to calculate the sample size. In the F-test for ANCOVA, effect size, alpha error probability, power, number of covariates, and number of groups were set 0.25, 0.05, 0.95, 4, and 5, respectively. The total sample size and actual power were 400 and 0.950. For the Z-tests for logistic regression, tails, odds ratio, mull hypothesis, alpha error probability, power, X distribution, X parm π were set to two, 2.5, 0.20, 0.05, 0.95, binomial, and 0.5, respectively. The total sample size and actual power were found to be 315 and 0.950, respectively. Therefore, the sample size of this study was confirmed to be sufficient.

## Results

### Participant characteristics

Table 1 shows participant characteristics. Among 895 participants, the mean age of 62.32 years (standard deviation: SD = 11.19) in 422 men was not significantly different from that of 62.91 years (SD = 11.41) in 473 women. The percentages of men who were current drinkers (*p* < 0.001), current smokers (*p* < 0.001), with hypertension (*p* = 0.012), and with diabetes (*p* = 0.004) were significantly higher than those of women. Triglycerides (*p* < 0.001), total energy (*p* < 0.001), zinc (*p* < 0.001), copper (*p* = 0.037), and manganese (*p* = 0.001) were significantly higher in men that in women. The percentage of participants with CAD did not significantly differ by sex.

**Table 1.** Participant Characteristics

	Men (*n* = 422)		Women (*n* = 473)		*p*-value^a^
	Mean/n	SD/%	Mean/n	SD/%	
Age, years	62.32	11.19	62.91	11.41	0.819
BMI, kg/m^2^	23.93	3.11	22.72	3.26	0.376
Current drinker, n (%)	305	72.27	117	24.74	<0.001
Current smoker, n (%)	349	82.70	70	14.80	<0.001
Hypertension, n (%)	145	34.4	126	26.6	0.012
Diabetes, n (%)	50	11.8	30	6.3	0.004
TG, mg/dL	131.50	87.50	106.50	56.07	<0.001
HDLC, mg/dL	60.82	16.03	68.50	17.01	0.053
LDLC, mg/dL	119.75	32.96	128.36	29.65	0.213
DL, n (%)	192	45.50	228	48.20	0.418
CAD, n (%)	20	4.74	18	3.81	0.489
Total energy, kcal	2084.70	604.45	1666.15	518.03	<.001
Zinc, mg/day	8.91	3.01	7.74	2.65	<0.001
Copper, mg/day	1.27	0.42	1.12	0.40	0.037
Manganese, mg/day	3.25	1.22	2.79	1.03	0.001

a The variables of means and SD are the Student’s *t*-test, and those of n and percent are chi-square tests. Abbreviations: BMI, body mass index; CAD, coronary artery disease; DL, dyslipidaemia; HDLC, high-density lipoprotein cholesterol; LDLC, low-density lipoprotein cholesterol; SD, standard deviation; TG, triglycerides.

### Comparisons between normolipidaemic and dyslipidaemic groups

Table 2 shows the characteristics of the two lipidaemic groups. The mean age of 420 patients in the dyslipidaemic group (63.56 years) was significantly higher than that of 475 patients in the normolipidaemic group (61.80 years, *p* = 0.027). In the lipid assessment, triglyceride (*p* < 0.001) and LDLC (*p* < 0.001) levels were significantly higher in the dyslipidaemic group than in the normolipidaemic group. The percentage of participants with CAD and the intake of trace minerals did not significantly differ between the two lipidaemic groups.

**Table 2.** Comparisons between normolipidemic and dyslipidaemic groups

	NL (*n* = 475)		DL (*n* = 420)		*p*-value^a^
	Mean/*n*	SD/%	Mean/n	SD/%	
Age, years	61.80	11.65	63.56	10.84	0.027
Sex (Men), n (%)	230	48.42	192	45.70	0.418
BMI, kg/m^2^	22.68	3.08	23.97	3.29	0.196
Current drinker, n (%)	229	48.21	193	45.95	0.499
Current smoker, n (%)	236	49.68	183	43.57	0.063
Hypertension, n (%)	155	32.6	116	27.6	0.103
Diabetes, n (%)	48	10.1	32	7.6	0.193
TG, mg/dL	85.45	29.58	155.43	89.28	<0.001
HDLC, mg/dL	69.38	16.62	59.79	15.93	0.187
LDLC, mg/dL	109.31	18.70	141.25	34.40	<0.001
CAD, n (%)	18	3.80	20	4.80	0.471
Total energy, kcal	1864.51	605.94	1862.40	589.32	0.583
Zinc, mg/day	8.29	2.88	8.29	2.90	0.727
Copper, mg/day	1.19	0.42	1.19	0.41	0.561
Manganese, mg/day	3.00	1.20	3.02	1.08	0.053

a The variables of means and SD are the Student’s *t*-test, and those of n and percent are chi-square tests. Abbreviations: BMI, body mass index; CAD, coronary artery disease; DL, dyslipidaemia; HDLC, high-density lipoprotein cholesterol; LDLC, low-density lipoprotein cholesterol; NL, normolipidaemia; SD, standard deviation; TG, triglycerides.

### Comparison between non-CAD and CAD groups

Table 3 shows the characteristics of the two CAD groups. The mean age of 38 patients in the CAD group (72.61 years) was significantly higher than that of 857 patients in the non-CAD group (62.19 years, *p* = 0.031). The percentage of participants with hypertension was significantly higher in the CAD group than in the non-CAD group (*p* = 0.047). HDLC levels were significantly lower in the CAD group than in the non-CAD group (*p* = 0.009). Copper (*p* = 0.041) and manganese (*p* < 0.001) intakes were significantly higher in the CAD group than in the non-CAD group. The percentage of participants with DL did not significantly differ between the two CAD groups.

**Table 3.** Comparisons between non-CAD and CAD groups

	Non-CAD (*n* = 857)		CAD (*n* = 38)		*p*-value^a^
	Mean/*n*	SD/%	Mean/n	SD/%	
Age, years	62.19	11.22	72.61	8.35	0.031
Sex (male), n (%)	402	46.90	20	52.63	0.489
BMI, kg/m^2^	23.27	3.26	23.59	2.99	0.465
Current drinker, n (%)	409	47.72	13	34.20	0.102
Current smoker, n (%)	403	47.02	16	42.10	0.548
Hypertension, n (%)	254	29.6	17	44.7	0.047
Diabetes, n (%)	74	8.6	6	15.8	0.130
TG, mg/dL	117.68	73.29	132.00	80.64	0.999
HDLC, mg/dL	65.25	17.07	56.63	12.31	0.009
LDLC, mg/dL	0.30	0.46	0.24	0.43	0.380
DL, n (%)	400	46.67	20	52.63	0.471
Total energy, kcal	1866.59	593.58	1793.91	692.81	0.268
Zinc, mg/day	8.29	2.86	8.28	3.40	0.137
Copper, mg/day	1.19	0.41	1.23	0.51	0.041
Manganese, mg/day	3.00	1.13	3.20	1.51	<0.001

a The variables of means and SD are the Student’s *t*-test, and those of n and percent are chi-square tests. Abbreviations: BMI, body mass index; CAD, coronary artery disease; DL, dyslipidaemia; HDLC, high-density lipoprotein cholesterol; LDLC, low-density lipoprotein cholesterol; SD, standard deviation; TG, triglycerides.

### Interaction between DL and CAD with trace mineral intake

Table 4 shows the results of an analysis of the main effects and interactions of DL and CAD with trace mineral intake using a two-way ANCOVA. Covariates were adjusted for age, sex, BMI, current drinkers, and current smokers. The trace minerals that showed a main effect in the two lipidaemic groups were zinc (*p* = 0.008), copper (*p* = 0.022), and manganese (*p* = 0.002). No trace minerals showed a main effect in the two CAD groups. The trace minerals that showed an interaction between the lipidaemic and CAD groups were zinc (*p* = 0.004), copper (*p* = 0.010), and manganese (*p* < 0.001). In multiple comparisons of the dyslipidaemic group using the Bonferroni method, the intake of zinc (*p* = 0.010) and copper (*p* = 0.023) was significantly lower in the CAD group than in the non-CAD group. In contrast, no significant differences were observed in these two trace mineral intakes between the two CAD groups in the normolipidaemic group. Therefore, these results indicate that zinc and copper intake was significantly lower in the CAD group than in the non-CAD group in the dyslipidaemic group, but did not significantly differ in the normolipidaemic group.

**Table 4.** Interactions between DL and CAD with trace mineral intake

	NL (*n* = 475)				DL (*n* = 420)				*p*-value^a^		
	Non-CAD (*n* = 457)		CAD (*n* = 18)		Non-CAD (*n* = 400)		CAD (*n* = 20)		Main effect		Interaction
	EMM	95%CI (lower, upper)	EMM	95%CI (lower, upper)	EMM	95%CI (lower, upper)	EMM	95%CI (lower, upper)	*P*1	*P*2	*P*3
Zinc, mg/day	8.26	(8.00, 8.52)	9.29	(7.97, 10.60)	8.36	(8.08, 8.64)	6.69	(5.45, 7.94)	0.008	0.502	0.004
Copper, mg/day	1.18	(1.15, 1.22)	1.32	(1.13, 1.51)	1.20	(1.16, 1.24)	0.99	(0.81, 1.17)	0.022	0.572	0.010
Manganese, mg/day	2.97	(2.87, 3.07)	3.61	(3.09, 4.12)	3.04	(2.94, 3.15)	2.42	(1.94, 2.91)	0.002	0.972	<0.001

a Two-way ANCOVA. Covariates were adjusted for age, sex, BMI, current smokers, and current drinkers. *P*1: Lipidaemic groups, *P*2: CAD groups, *P*3: lipidaemic × CAD groups. Abbreviations: ANCOVA, analysis of covariance, BMI, body mass index; CAD, coronary artery disease; CI, confidence interval, DL, dyslipidaemia; EMM, estimated marginal means; NL, normolipidaemia.

### Logistic regression analysis of CAD with trace mineral intake stratified by DL

Table 5 shows the results of the multiple logistic regression stratified by normolipidaemic and dyslipidaemic groups, with the dependent variable of CAD. In model 1, covariates were adjusted for age, sex, BMI, current smokers, and current drinkers, with each trace mineral imputed individually as independent variables. Significant independent variables for CAD were zinc (OR: 0.752; 95% CI: 0.606, 0.934; *p* = 0.010), copper (OR: 0.175; 95% CI: 0.042, 0.726; *p* = 0.016), and manganese (OR: 0.494; 95% CI: 0.291, 0.839; *p* = 0.009) in the dyslipidaemic group. The Bonferroni correction divided by the number of individually input trace minerals showed that zinc and manganese were significant independent variables. There was no trace mineral for CAD in the normolipidaemic group.

**Table 5.** Multiple Logistic Regression Analysis

		Model 1					Model 2				
		B	*p*-value	OR	95%CI		B	*p*-value	OR	95%CI	
					Lower	Upper				Lower	Upper
NL	Zinc	0.116	0.147	1.123	0.960	1.314	0.112	0.159	1.119	0.957	1.309
(n = 475)	Copper	0.834	0.142	2.302	0.756	7.014	0.831	0.144	2.296	0.753	7.005
	Manganese	0.377	0.046	1.458	1.007	2.110	0.380	0.043	1.462	1.012	2.111
DL	Zinc	–0.285	0.010	0.752	0.606	0.934	–0.289	0.010	0.749	0.600	0.934
(n = 420)	Copper	–1.744	0.016	0.175	0.042	0.726	–1.779	0.015	0.169	0.040	0.713
	Manganese	–0.704	0.009	0.494	0.291	0.839	–0.717	0.009	0.488	0.285	0.835

The dependent variable is CAD. Model 1: Covariates were adjusted for age, sex, BMI, current smokers, and current drinkers. Model 2: Covariates in model 1 plus hypertension and diabetes. Abbreviations: B, partial regression coefficient; CI, confidence interval; DL, dyslipidaemia; NL, normolipidaemia; OR, odds ratio.

Model 2 was the same analysis as model 1, with the addition of hypertension and diabetes as covariates. Significant independent variables in the dyslipidaemic group were zinc (OR: 0.749; 95% CI: 0.600, 0.934; *p* = 0.010), copper (OR: 0.169; 95% CI: 0.040, 0.713; *p* = 0.015), and manganese (OR: 0.488; 95% CI: 0.285, 0.835; *p* = 0.009). In the Bonferroni correction for the number of items, zinc and manganese were significant independent variables. In the normolipidaemic group, trace minerals other than manganese were not independent variables for CAD. Therefore, low zinc and manganese intake inversely correlated with CAD in the dyslipidaemic group, but not in the normolipidaemic group.

## Discussion

The present results demonstrated that zinc intake was significantly lower in the CAD group than in the non-CAD group in the dyslipidaemic group, but not in the normolipidaemic group in multiple comparisons by the Bonferroni method after a two-way ANCOVA or in the multiple logistic regression analysis.

A review by Mihăilă^(22)^ that examined the relationship between DL and CAD revealed that HDLC, the TC/HDLC ratio, and TG/HDLC ratio were strongly related in a population with cardiovascular risk factors. Andreadou *et al.* ^(23)^ showed that patients with hypercholesterolemia were predisposed to myocardial dysfunction and myocardial infarction through increased oxidative stress, mitochondrial dysfunction, and apoptosis induced by inflammation. The present study revealed that HDLC was significantly lower in the CAD group than in the non-CAD group. Previous reviews^(27,28)^ on the relationship between HDLC and CAD reported that low HDLC increased the risk of CAD. In contrast, the present results showed no increase in TG or LDLC in the CAD group. Although the underlying reason is unclear, dietary habits represented by the Japanese diet may be involved because we targeted middle-aged and older adults in a rural Japanese community.

The present results on zinc, copper, and manganese showed that its lower intake in the dyslipidaemic group was associated with CAD after adjustments for age, sex, BMI, current smokers, and current drinkers. A case–control study by Ghayour-Mobarhan *et al.* ^(29)^ showed that dyslipidaemic patients with CAD had a lower zinc intake and zinc/copper ratio than patients without CAD or healthy university/hospital personnel as controls. It has been reported that lower zinc intake is associated with CAD^(29)^ and CAD mortality.^(13)^ Furthermore, a systematic review by Ranasinghe *et al.* ^(11)^ revealed that zinc supplementation significantly reduced TC, LDLC, and TG. Therefore, improvements in HDLC and LDLC reduce the risk of CAD, indicating that the high zinc intake in the dyslipidaemic group decreased the risk of developing CAD. A reason why the low zinc intake was associated with CAD in the dyslipidaemic group only may be attributed to an insufficient trace mineral intake, which did not improve blood lipids and, thus, failed to reduce the risk of developing CAD. Alternatively, copper^(14,15)^ and manganese^(9)^ have been associated with a decrease in serum lipids. In our multiple comparisons using the Bonferroni method, zinc and copper correlated with CAD in the dyslipidaemic group only. Additionally, in multiple logistic regression analyses using two different models, zinc and manganese were significant independent variables in the dyslipidaemic group after the Bonferroni correction. Therefore, a novel result in the present study is that a low zinc intake with DL was strongly associated with CAD in the simultaneous evaluation of several trace minerals. As a possible mechanism, the combination of low zinc intake increasing total cholesterol, LDLC, and triglycerides,^(6)^ as well as low copper intake decreasing HDLC^(14)^ and low manganese intake increasing CAD risk,^(9)^ suggests that low intake of these trace minerals is comprehensively associated with CAD only in the DL group.

Typical comorbidities related to CAD include hypertension^(30,31)^ and diabetes.^(32,33)^ A systematic review and meta-analysis by Nicoll *et al.* ^(33)^ demonstrated the importance of hypertension and diabetes as predictors of the presence and extent of coronary artery calcification. Even in our multiple logistic regression analysis with hypertension and diabetes as covariates in addition to lifestyle, zinc was a significant independent variable that negatively correlated with CAD in the dyslipidaemic group. Additionally, zinc has been shown to improve insulin sensitivity.^(34,35)^ Therefore, another novel result of the present study is that a high zinc intake was identified as a beneficial factor for CAD, even in the presence of diabetes and hypertension comorbidities in addition to DL.

Among DL, FH is a common hereditary autosomal dominant disorder characterized by high plasma cholesterol levels, with an estimated frequency of 1 in 200.^(36)^ The most common causes of FH are pathogenic variants of the LDL receptor (*LDLR*) gene, as well as deleterious mutations in the apolipoprotein B (*APOB*) gene decreasing the binding of LDL to the *LDLR* and gain-of-function mutations in the gene for proprotein convertase subtilisin/kexin 9 (*PCSK9*) resulting in the increased destruction of *LDLR*.^(37)^ The presence of *APOB* or *PCSK9* mutations is included in the diagnostic criteria of the Simon Broome Criteria for the Diagnosis of FH. A genetic analysis by Koyama *et al.* ^(38)^ revealed that rs564427867 in *PCSK9* and rs879255211 in *LDLR* were associated with elevated serum levels of TC in the Japanese population. Since hereditary hypercholesterolemia may not be associated with trace mineral intake or serum lipid levels, we excluded residents with rs564427867 or rs879255211 risk alleles from the statistical analyses.

The limitations of the present study ought to be addressed. First, since this was a cross-sectional study, the causal relationship between trace minerals, serum lipids, and CAD cannot be elucidated. Secondly, comorbidities other than DL were self-reported by a questionnaire and not diagnosed by data. Thirdly, trace mineral intake by the BDHQ may lack objectivity and does not include supplement intake, Fourthly, due to the small number of CAD participants, further analysis with a larger sample size is needed. Fifthly, we did not examine the trace mineral intake from living environments. Sixthly, we did not evaluate the trace minerals in serum. Finally, since the results are from one rural town, it may not be representative of Japan.

## Conclusions

Results of the present study showed that in the dyslipidaemic group, trace mineral intake was significantly lower in the CAD group than in the non-CAD group, whereas in the normolipidaemic group, this association was not observed between the two CAD groups. Further longitudinal studies are needed to confirm this relationship.

## Data Availability

Data in the present study are available upon request from the corresponding author. Data are not publicly available due to privacy and ethical policies.
